# Dynamic transcriptome analysis provides molecular insights into underground floral differentiation in *Adonis Amurensis* Regel & Radde

**DOI:** 10.1186/s12863-024-01220-2

**Published:** 2024-03-21

**Authors:** Hui Xin, Lifan Zhang, Hongtao Wang, Xingzun Zhu

**Affiliations:** 1 School of Landscape Architecture, Changchun University, 6543 Weixing Road, Changchun, China; 2 College of Life Sciences, Tonghua Normal University, 950, Yucai Road, Tonghua, China

**Keywords:** *Adonis amurensis*, Flower differentiation, Transcriptome analysis, Underground flower development, Differentially expressed gene, Transcription factor, Plant hormones

## Abstract

Understanding flower developmental processes is a prerequisite for improving flowering ‘plants’ production. *Adonis amurensis* is a fascinating spring ephemeral plant that develops its flower organs underground. Nevertheless, knowledge of the molecular mechanisms driving this particular process is scarce. Herein, we examined transcriptional changes during underground flower differentiation in *A. amurensis* and unveiled key differently regulated genes and pathways. High-throughput RNA sequencing of meristems at different flower developmental stages, including flower primordium (FP), sepal stage (SE), perianth primordium (PE), stamen stage (ST), and pistil stage (PI), identified 303,234 unigenes that showed 44.79% similarity with sequences in *Aquilegia coerulea*. Correlations, principal component, and differentially expressed genes (DEGs) analyses revealed that few molecular changes occurred during the transition from PE to ST. Many DEGs exhibited stage-specific regulations. Transcription factor (TF) and phytohormone family genes are critical regulators of the floral differentiation process in *A. amurensis*. The most differentially regulated TFs were MADS, FAR1, MYBs, AP2/ERF, B3, C2H2, and LOBs. We filtered out 186 candidate genes for future functional studies, including 18 flowering/circadian-related, 32 phytohormone-related, and TF family genes. Our findings deepen our understanding of the underground flower differentiation process and offer critical resources to dissect its regulatory network in *A. amurensis*. These findings establish a foundational platform for researchers dedicated to exploring the unique phenotypic characteristics of this specific flowering modality and delving into the intricate molecular mechanisms underpinning its regulation and expression.

**Supplementary Information:**

The online version contains supplementary material available at 10.1186/s12863-024-01220-2.

## Introduction

Reproduction is the key determinant of species survival. In Angiosperm plants, flowering is one of the major developmental switches that result in the vegetative to the reproductive stage and involves complex genetic and epigenetic reprogramming to guarantee successful progeny [1–5]. The flower development process, including the establishment of floral meristems, formation of unique organ identities, and floral ‘structures’ differentiation, is regulated by a complex molecular network regrouping specific genes, hormones, and transcription factors that integrate endogenous signals and various environmental cues so that flowering takes place at the proper time [1, 6–10]. The regulation of flower differentiation incorporates multilevel gene expression patterns that occur in cells of inflorescence meristems, sepals, petals, stamens, and carpels primordium [9]. A wide diversity is observed in the flower development process among different species and varieties. Therefore, understanding the multilevel genetic regulatory network of flowering processes is a prerequisite to optimizing crop and important ornamental ‘plants’ production under this natural climate change.

In *Arabidopsis thaliana*, tremendous studies on the photoperiod, ambient temperature, autonomous, gibberellin, age-dependent, circadian clock, vernalization, and trehalose-6-phosphate pathway led to the identification of several regulatory elements of flowering induction [11, 12]. The regulators involved in the six main flower regulation pathways, including endogenous (autonomous, age, and gibberellin) and environmental (photoperiod, vernalization, and temperature) coordinate ‘signals’ transduction and gene interactions, via the transcriptional control of the expression of two floral pathway integrators, *FLOWERING LOCUS T* (*FT*) and *SUPPRESSOR OF OVEREXPRESSION OF CONSTANS 1* (*SOC1*) [13]. The study by Jang et al. demonstrated that *FT* regulation is likely species-specific, enabling each species to flower under favorable environmental conditions [12, 14]. Therefore, it is necessary to dissect the molecular regulatory network of flowering in each species, specifically in species with particular flower morphogenesis processes.

*Adonis amurensis* Regel & Radde is an important early spring short-lived herbaceous flower plant member of the Ranunculaceae family. It is principally distributed in Northeastern China, Korea, and Japan, where its organs are widely used as a traditional folk medicine to treat heart palpitations, epilepsy, and edema [15, 16]. The sexual reproduction process of early spring short-lived plants includes a long underground development process of flower organs and rapid blossoming after overwintering [17]. *A. amurensis* has a concise aboveground growth cycle and quickly completes flowering, pollination, and fertilization under low temperatures (day/night temperatures between − 15 and 10 °C) before the ice and snow melts in the spring in northeast China [18, 19]. Previous studies have identified key transcription factor family genes involved in flowering time regulation in *A. amurensis* [18–20]. These investigations provided fundamental and crucial insights into the transcriptional dynamics within the floral tissues of *Adonis amurensis*. The comprehensive analysis delineated the expression profiles of genes during the pivotal stages of floral development, contributing significantly to our understanding of the molecular underpinnings governing the phenotypic characteristics of this species. The distinctive life cycle of *Adonis amurensis*, particularly focusing on its unique underground developmental phase, notably diverges from the conventional flowering triggers observed in other plant species. Unlike most flora that rely heavily on aboveground environmental cues such as photoperiod and vernalization to initiate flowering, *A. amurensis* exhibits a remarkable subterranean phase of floral differentiation [21]. This phase occurs independently of the traditional stimuli, underscoring the need to elucidate the molecular frameworks and genetic regulation governing this atypical flowering process [22, 23]. Understanding these unique life cycle attributes sheds light on the adaptive strategies of *A. amurensis* and potentially unveils new pathways and mechanisms in plant developmental biology. ‘Hence, insight into the molecular mechanisms governing floral differentiation in *A. amurensis* and identifying the genetic regulators for functional studies and molecular breeding purposes is of significant interest.

The present study comprehensively analyzed the transcriptional changes that occur during flower differentiation in *A. amurensis* through high-throughput transcriptomics analysis of meristems at five developmental stages, including flower primordium, sepal stage, perianth primordium, stamen stage, and the pistil stage. Our results will facilitate the dissection of the genetic regulatory network of the underground flower differentiation process.

## Materials and methods

### Plant materials and characterization of *A. amurensis* flower organogenesis

In mid-May 2020, after the aboveground part of the plants withered, about 300 roots were dug from the Tuodaoling region, China: Longitude 125° 55′ 45″ E to 125° 35′ 59″ E; Latitude: 41° 37′ 55″ N to 41° 37′ 59″ N and transplanted to a practical cultivation base located at Tonghua Normal University, China: Longitude: 125° 58′ 49.63″ E; Latitude: 41° 44′ 47.04″ N). No permission is required to collect such wild samples. The plant materials were identified by Prof. Wang Hongtao, and kept at the publicly accessible herbarium. The detailed information is as follows. Herbarium, School of Life Sciences in Tonghua Normal University. Specimen No. THUN—MGK—03 − 001. To monitor subtle changes and short-term variations every three days from 10 May to 15 November 2020, the underground storage organs were dug out and brought back to the laboratory for washing. Then, the buds of the stored flower organs were carefully separated and fixed in the Formaldehyde Alcohol Acetic Acid (FAA), 10%:50%:5% + 35% water) fixation solution for more than 48 h to observe the developmental morphology of the flower organs. Supplementary observations were conducted from 10 May to 15 November 2021. The morphology of the buds was examined using scanning electron microscopy (SEM, S-3000 N, Hitachi Co., Ltd., Matsuda, Japan) following the same method as Wang et al. [17].

### Sample Collection for transcriptome sequencing

Based on the results of flower organogenesis observations, meristem samples in bulbs were collected in 2021 at five stages of the underground flower differentiation, including flower primordium, sepal, perianth, stamen, and pistil differentiation stages. Three biological repeats were sampled at each stage, frozen in liquid nitrogen, and kept at -80 °C until the total RNA extraction.

### RNA isolation, cDNA library construction, de novo assembly, DEGs analysis, and transcription factors (TFs) identification

TRIzol reagent (TaKaRa, China) was used to extract the total RNA from the meristem samples following the manufacturer’s instructions. It was treated with RNAse-free DNase I (Takara Bio Lnc, USA) to purify the RNA from DNA traces further. The extracted RNA quality was assessed using agarose gel Agilent 2100 Bioanalyzer system (Agilent Technologies, CA, USA), respectively (data not shown). NEBNext® UltraTM RNA Library Prep Kit for Illumina® (NEB, USA) was used to construct pair-end sequencing libraries, following the manufacturer’s instructions. Illumina HiSeq platform was used for RNA sequencing. The RNA sequencing was performed by Novogene (https://en.novogene.com/), as we have recently reported [17]. The raw data was filtered to ensure the data analysis’s quality and reliability. Low-quality and short sequence reads (< 50 bp) were removed using FastQC and in-house Perl scripts. After that, the high-quality clean data were used to perform de novo assembly. The clean data’s Q20, Q30, GC-content and sequence duplication level were also calculated. Transcriptome assembly was accomplished based on the remaining high-quality clean data using Trinity v.2.6.6 [24].

### Functional annotation of DEGs

The mapped reads numbers were calculated using featureCounts v1.5.0-p3 [25]. Then, calculating the expected number of fragments per kilobase of exon model per million reads mapped (FPKM) of each gene based on the length of each gene and reads count mapped to the gene. Differentially expressed genes (DEGs) among the samples from different floral developmental stages were identified using the DESeq R package (v1.18.0) [48]. The thresholds for DEGs identification were|log_2_Fold Change| ≥ 1 and *p*-value < 0.05. We functionally annotated the DEGs via BlastX (E-value > 10 − 5) against the Protein family (Pfam) database, Swiss Prot, NCBI non-redundant (Nr) databases, Cluster of Orthologous Groups databases (COG/KOG), Kyoto Encyclopedia of Genes and Genomes pathway database (KEGG), Gene Ontology (GO), and Trembl. The GO and KEGG pathway enrichment analyses were carried out using Blast2GO [26] and KOBAS2.0 [27] programs, respectively. Significantly enriched GO terms or KEGG pathways were screened at a *p* or q value of ≤ 0.05.

### Quantitative real-time PCR (qRT-PCR) analysis

Nine DEGs were randomly selected for qRT-PCR analysis to verify the reliability of the RNA-seq data. The analysis was conducted on Light Cycler 480 (Roche, Switzerland) real-time PCR system, with ChamQ™ SYBR1 qPCR Master Mix (Vazyme Biotech, Nanjing, China). The *Actin8* gene was used as the internal control for transcript normalization. Three biological replicates were achieved for each gene, and the 2^-ΔΔCT^ method was applied [28]. The list of the selected genes and their specific primers is presented in Table S9.

### Statistical analysis

The prcomp and corr functions in R were used for Principal Component Analysis (PCA) and correlation analysis, respectively. GraphPad Prism v9.0.0121 (GraphPad 159 Software Inc., La Jolla, CA, USA) was used to construct bar graphs and pies. TBtools software was used for gene expression profile heatmap construction and qRT-PCR data analysis [29].

**Fig. 1:**
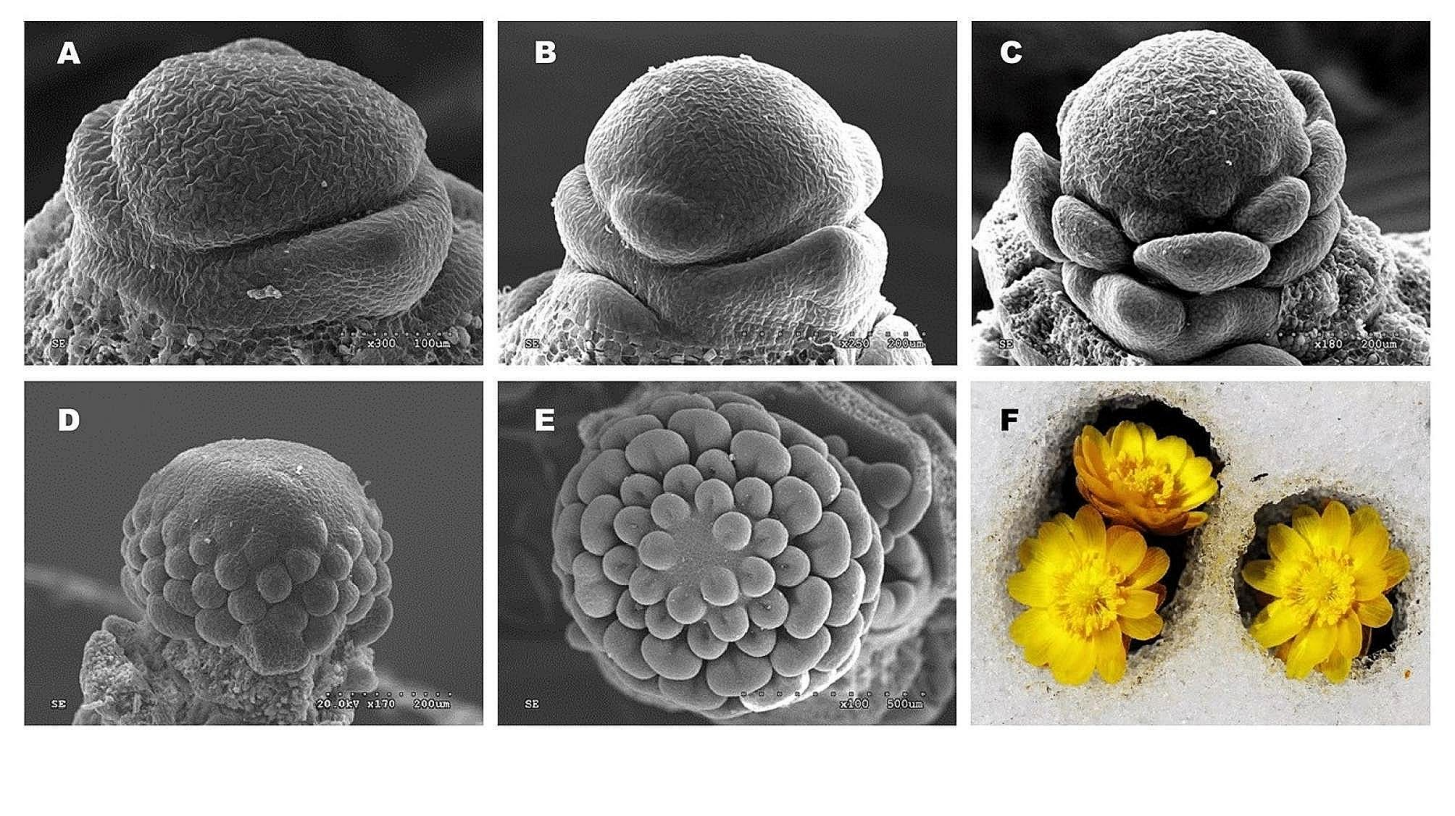
Overview of *A. amurensis* flower organogenesis. (**A-E**) Scanning electronic microscopic (SEM) images of *A. amurensis* developing flower at flower primordium (FP), sepal (SE), petal (PE), stamen (ST), and pistil (PI) differentiation stages, respectively. (**F**) An image of *A. amurensis* flower during early spring

## Results

### Phenology of flower organogenesis in *A. amurensis*

Through continuous sampling and observation, we found that the rhizome of *A. amurensis* began to differentiate into new roots and underground buds on April 20 to July 2023 (the flowering stage). Bud ‘scales’ differentiation took place from full flowering to the withering of aboveground plants (April 20 ∼ May 28). Then, the rhizome entered dormancy, and the underground buds began switching from vegetative to reproductive growth. The flower organogenesis process could be divided into five stages, including flower primordium differentiation (May 25 ∼ June 10), sepal differentiation (June 11 ∼ June 18), petal differentiation (June 19 ∼ June 28), stamen differentiation (June 29 ∼ July 15), and pistil differentiation (July 16 ∼ July 23) (Fig. 1). The whole process of flower organogenesis lasted less than two months.

### Dynamic transcriptome profiles of *A. amurensis* flower during development

To provide insight into the underground flower development process in *A. amurensis*, meristem samples were collected at different flower development stages, including flower primordium (FP), sepal stage (SE), perianth primordium (PE), stamen stage (ST), and pistil stage (PI) (Fig. 1) and subjected to transcriptome sequencing and analysis. The RNA sequencing yielded 53,848,828–90,034,816 bp of raw reads, with clean reads ranging from 49,674,888 to 85,680,246 bp (Table S1). The error rate of all samples was 0.03%, and the GC content varied from 44.33 to 44.79 (Table S1). The Q20 and Q30 values for all samples ranged from 98.02 to 98.49% and 93.04 to 94.68%, respectively, supporting the high quality of the sequencing data. Therefore, we de novo assembled the clean reads into 303,234 unigenes using the Trinity software [24, 30]. The N50 value of transcripts and unigenes was 1,106 and 1,125, respectively, with an average length of with an average length of 743 and 763 bp, respectively (Figure S1). Sequence similarity analysis indicated that 44.79% of unigenes are identical to sequences in *Aquilegia coerulea*, the same family member species (Figure S2B). Annotation analyses indicated that 20.49, 16.63, 29.42, 29.15, 20.78, 16.48, and 15.73% unigenes were highly similar to known proteins the KEGG, Swiss-Prot, NR, Trembl, GO, KOG, and Pfam databases, respectively (Figure S2A).

**Fig. 2:**
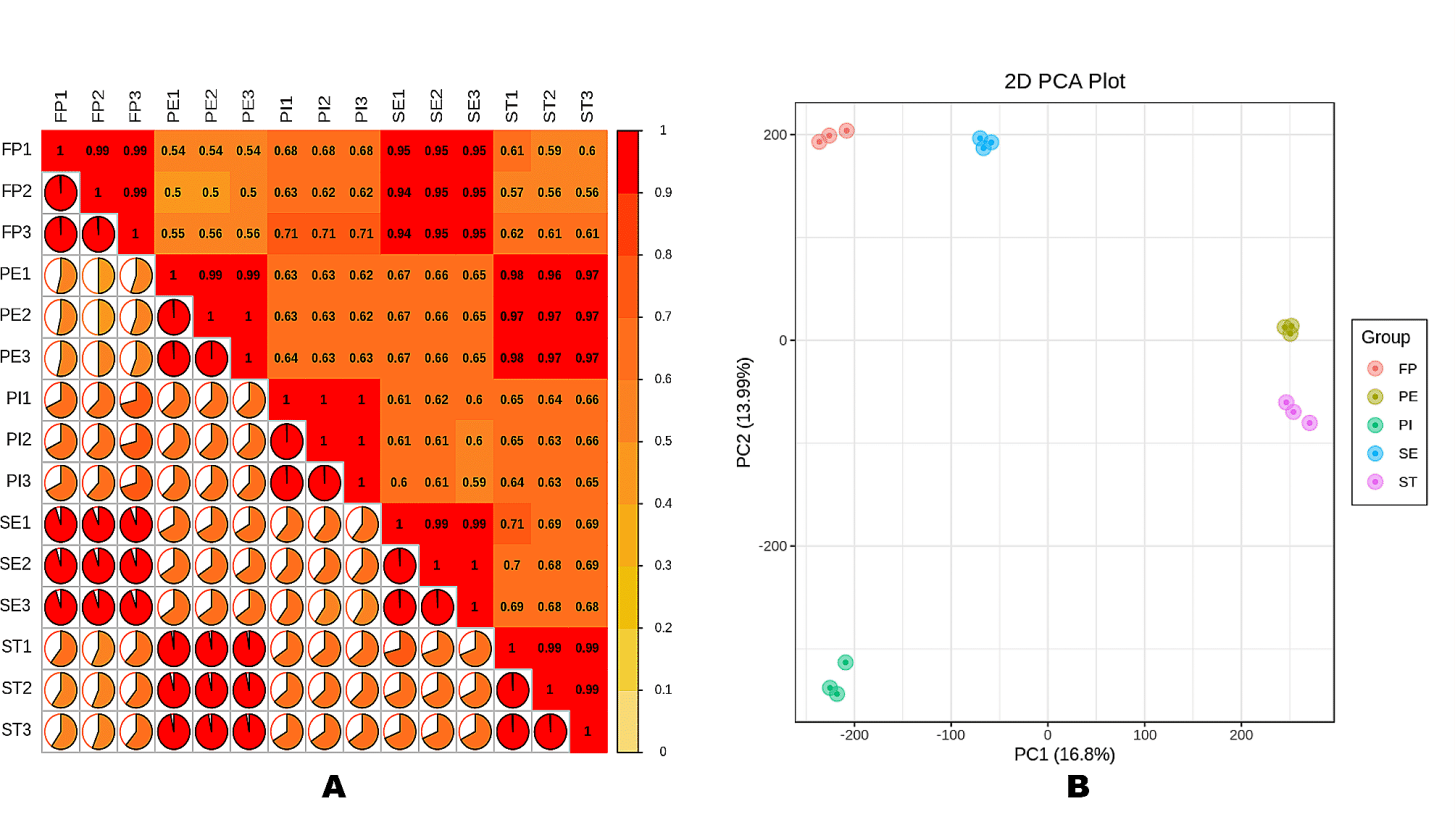
Correlation analysis (**A**) and principal component analysis (**B**) of *A. amurensis* of the different samples based on the FPKM values

Correlations analysis revealed strong correlations (*r* > 0.99) between samples of the same group (Fig. 2A), indicating the reproducibility of the experiment. As shown in Fig. 2A, strong correlations were also observed between FP vs. SE samples (*r* > 0.94) and between PE and ST samples (*r* > 0.97), suggesting major transcriptome changes did not occur during the transitions FP to SE, and PE to ST, respectively. PCA supported the correlation analysis results (Fig. 2B). Samples from the same group were clustered in the PCA plot, confirming the experiment is reliable and repeatable. PI samples were completely separated from other groups (Fig. 2B), suggesting major transcriptome regulations might have occurred during the transition from ST to PI.

### Differentially expressed genes (DEGs) during flower development in *A. amurensis*

**Fig. 3:**
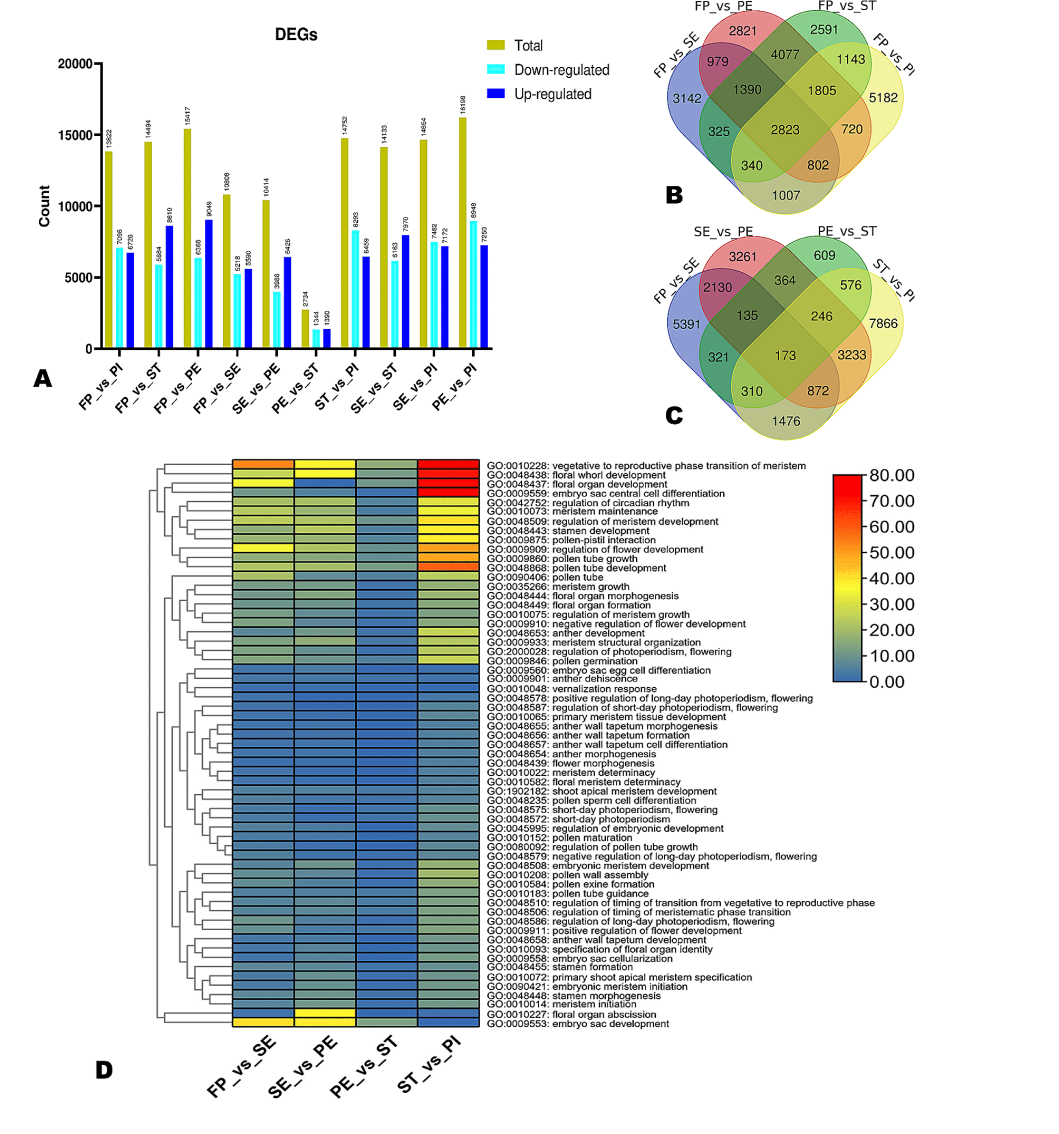
Differentially expressed genes (DEGs) between groups and key enriched pathways related to circadian/flowering. (**A**) Number of DEGs in the pairwise comparison between groups. (**B**) The Venn diagram shows the number of common DEGs in the pairwise comparison of other development stages against PF. (**C**) The Venn diagram shows the common number of common DEGs when comparing consecutive developmental stages. (**D**) Heatmap of significantly enriched GO terms related to circadian and flowering. The redder the color, the greater the number of genes are enriched

To enable an overview of transcriptional changes that occur during flower organs’ development in *A. amurensis*, we performed pairwise analyses of DEGs and filtered out all DEGs applying the criteria of *p*-value < 0.05 and threshold|log_2_ fold Change| ≥ 1. Different groups have shown different numbers of DEGs and their up and down-regulation. In the group FP vs. PI, a total of 13,822 genes were differentially expressed, out of which 7096 were up-regulated while 6726 were down-regulated. In the group FP vs. ST, a total of 14,494 genes were deferentially expressed, out of which 5884 were down-regulated while 8610 were up-regulated. In the group FP vs. PE, a total of 15,417 genes were differentially expressed, out of which 6368 were downregulated while 9049 were up-regulated. In the group FP vs. SE, a total of 10,808 genes were differentially expressed, out of which 5218 were downregulated while 5590 were up-regulated. In the group SE vs. PE, a total of 10,414 genes were differentially expressed, out of which 3988 were downregulated while 6426 were up-regulated. In the group PE vs. ST, a total of 2734 genes were differentially expressed, out of which 1344 were downregulated while 1390 were up-regulated. In the group ST vs. PI, a total of 14,752 genes were differentially expressed, out of which 8293 were downregulated while 6459 were up-regulated. In the group SE vs. ST, a total of 14,133 genes were differentially expressed, out of which 6163 were downregulated while 7970 were up-regulated. In the group SE vs. PI, a total of 14,654 genes were differentially expressed, out of which 7482 were downregulated while 7172 were up-regulated. Lastly, in the group PI vs. PE, a total of 16,198 genes were differentially expressed, out of which 8984 were downregulated while 7250 were up-regulated (Fig. 3A). When comparing consecutive time points, we found that the DEGs number decreased from FP to ST, then increased at PI (Fig. 3A). For instance, there were 10,808 (5,590 up-regulated), 10,414 (6,426 up-regulated), 2,734 (1,390 up-regulated), and 14,752 (6,459 up-regulated) DEGs between FP vs. SE, SE and PE, PE and ST, and ST and PI, respectively. A Venn diagram was generated to show the number of common DEGs in the pairwise comparison of other development stages against PF (Fig. 3B). The volcano plots are shown in Figure S3. Of these DEGs, only 173 were common (Fig. 3C), indicating that many genes are stage-specific regulated during flower development in *A. amurensis*. The stage-specific regulation of DEGs was supported by K-means analysis. The clusters are provided in Figure S4, while the detailed description is provided in Table S2).

We selected DEGs in the pairwise comparison between consecutive time points for functional annotations to figure out the main pathways differentially regulated during flower development in *A. amurensis*. The results showed that the DEGs were mainly involved in the biosynthesis of secondary metabolites, protein processing in the endoplasmic reticulum, plant hormone signal transduction, starch and sucrose metabolism, ABA transporters, and MAPK signaling pathway (Figures S5 and S6; Tables S3-S6). We filtered out flowering-related GO enrichment terms to allow an overview of the regulation of flowering-related genes during flower ‘organs’ development (Fig. 3D). The result confirmed more genes are differentially regulated during the transition from ST to PI, and no major transcriptional changes have occurred during the transition from PE to ST. The most enriched flowering-related GO terms were vegetative to the reproductive phase transition of the meristem, flower whorl development, flower organ development, embryo sac central cell differentiation, pollen tube growth and development, and regulation of flower development (Fig. 3D).

**Fig. 4:**
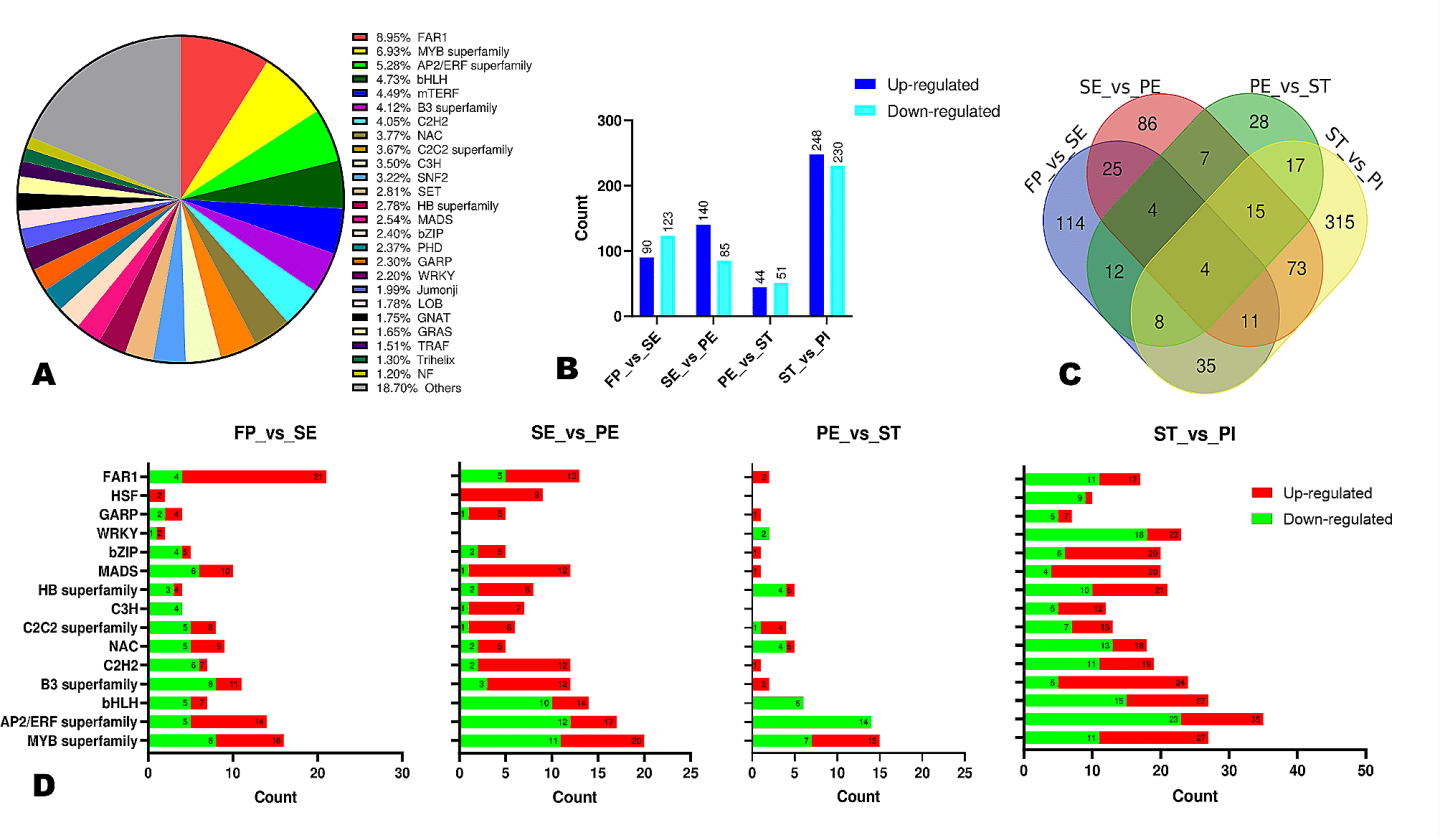
Transcription factors (TFs) involved in floral differentiation in *A. amurensis*. (**A**) TF families are identified in the transcriptome of *A. amurensis* during flower organogenesis. (**B**) Numbers of differentially expressed TFs (DETFs) in pairwise comparison between consecutive developmental stages. (**C**) Venn diagram illustrating common number of DETFs. (**D**) Overview of numbers of up- and down-regulated DEGs belonging to major identified TF families

### Transcription factors (TFs) involved in flower development in *A. amurensis*

TFs play essential roles in regulating plant growth and developmental processes. We identified in total 2,915 unigenes encoding diverse TF family genes. The major classes of TFs were FAR1, MYBs, AP2/ERF, bHLH, mTERF, B3, C2H2, NAC, and C2C2 (Fig. 4A). MADS, WRKY, and GRAS accounted for 2.54, 2.20, and 1.65%, respectively. We screened out TFs among the DEGs and identified 213 (90 up-regulated), 225 (140 up-regulated), 95 (44 up-regulated), and 478 (248 up-regulated) DEGs encoding TFs between FP vs. SE, SE and PE, PE and ST, and ST and PI, respectively (Fig. 4B). Venn diagram showed that only four TFs were differentially expressed along with the flower development (Fig. 4C). The list of the four genes is provided in Table S7. Figure 4D presents the number of up-and down-regulated DEGs belonging to the major groups of TFs in *A. amurensis.* The results indicated that MADS, FAR1, MYBs, AP2/ERF, B3, C2H2, and HSF TF family genes might play key regulatory functions during *A. amurensis* flower differentiation.

**Fig. 5:**
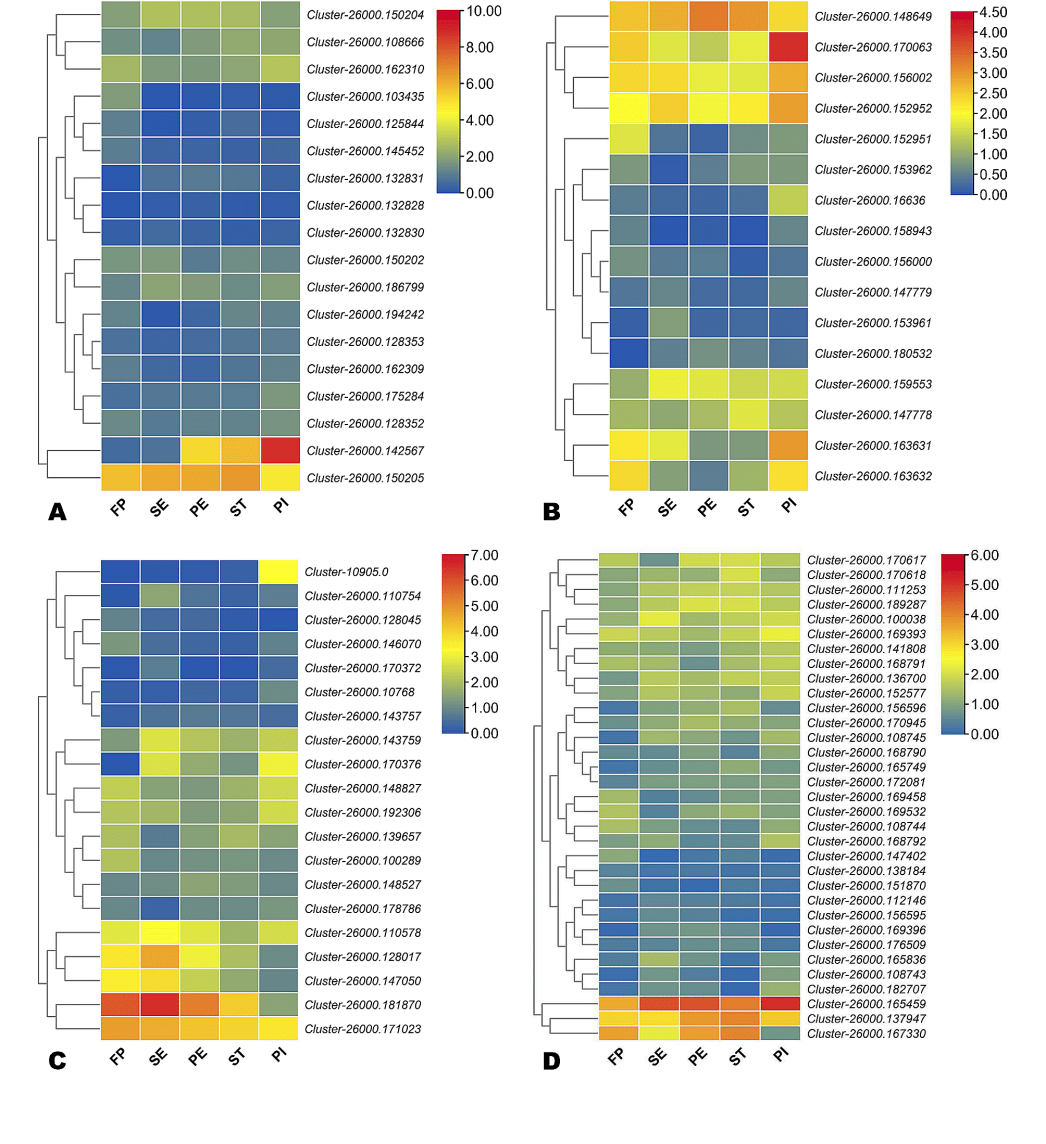
Heatmap of log_2_ FC values of the DEGs belonging to MADS (**A**), DELLA (**B**), MYB (**C**), and FAR1 (**D**) families

We then examined the expression profiles of differentially expressed MADS, GRAS (DELLA subfamily), MYB, FAR1, AP2/ERF, and LOB family genes (Fig. 5 and S7). The results showed that most TF family genes were stage-specific up-regulated or down-regulated, confirming they may play essential roles in the regulation network of flower differentiation in *A. amurensis.* For instance, the gene *Cluster-26000.154977* (AP2) was significantly up-regulated (|log_2_ Fold Change| > 7.8) at SE, PE, and PI stages. The genes *Cluster-26000.18804* (bHLH) and *Cluster-26000.162102* (MYB) were significantly up-regulated (|log_2_ Fold Change| > 8.6), specifically at the PI stage. The genes *Cluster-26000.166133* and *Cluster-26000.154590* (PHD) were significantly up-regulated (|log_2_ Fold Change| > 6.6) at SE and PE stages. The genes *Cluster-26000.141313*, *Cluster-26000.147835*, and *Cluster-26000.149428* (WRKY) were significantly down-regulated (|log_2_ Fold Change| > 3.05) specifically at PI stage.

### Phytohormone, circadian, and flowering-related DEGs

**Fig. 6:**
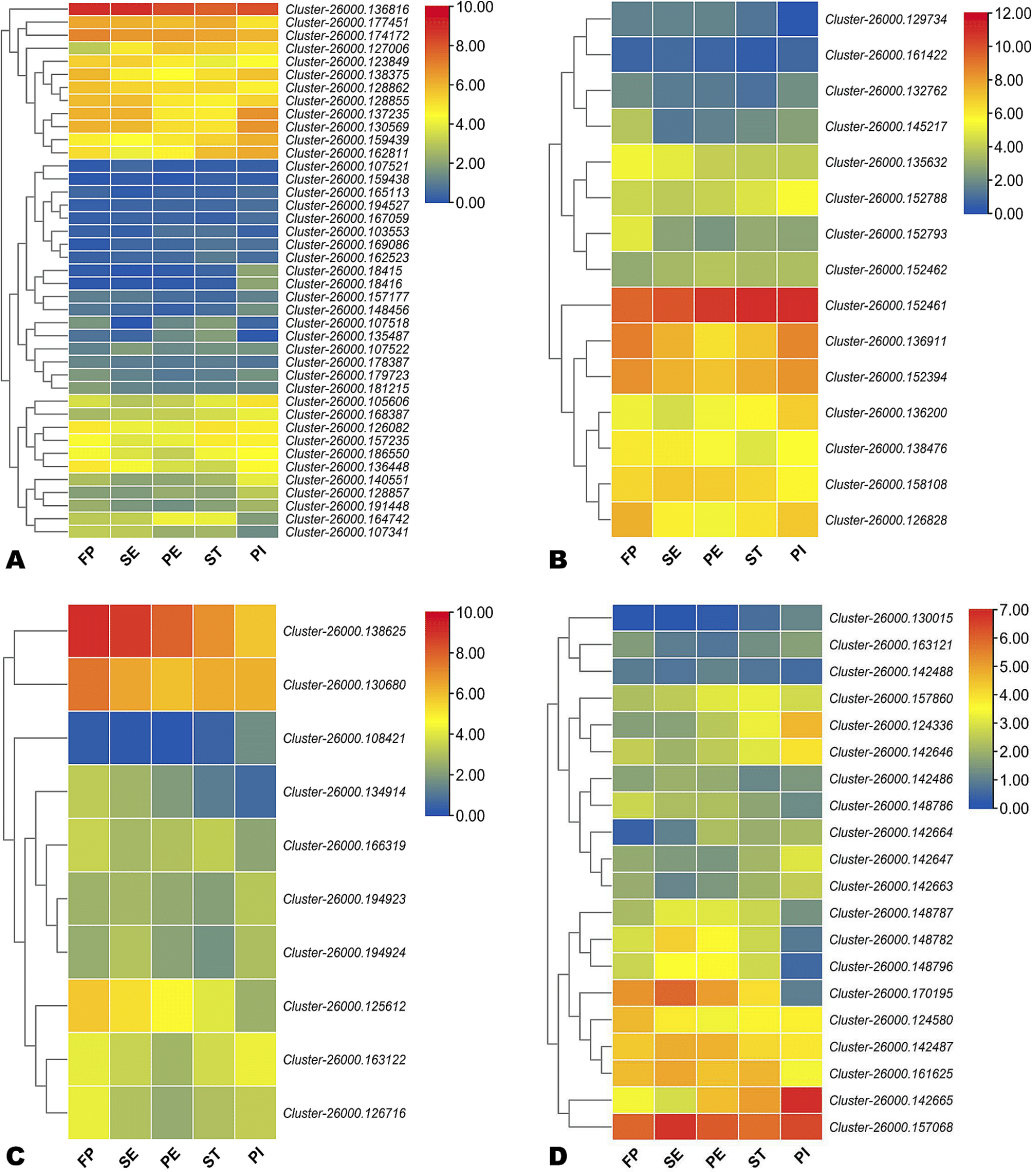
Heatmap of log_2_ FC values of phytohormone-related DEGs. (**A**) Auxins. (**B**) Gibberellin. (**C**) Jasmonic acid. (D) Salicylic acid

Phytohormones are critical for the coordinated initiation and development of flowers. Figure 6 and S8 show that many phytohormone-related genes were differentially regulated during *A. amurensis* flower development. We identified 41, 31, 20, 16, 15, 10, and 6 auxins, brassinosteroid, salicylic acid, ABA, gibberellin, jasmonic acid, and cytokinin-related DEGs, respectively (Fig. 6 and S8). Most of these genes were up-regulated at least at one stage of *A. amurensis* flower differentiation. This indicates they might play important roles in modulating flower ‘organs’ formation and transducing environmental signals. The genes *Cluster-26000.166280* (Cytokinin-related), *Cluster-26000.131278* (ABA-related), and *Cluster-26000.138625* and *Cluster-26000.125612* (JA-related) were significantly induced at the FP stage, suggesting they might be essential for the transition from vegetative to reproductive tissues development. The genes *Cluster-26000.135983* (BR-related), *Cluster-26000.174328* and *Cluster-26000.138802* (Cyt-related), and *Cluster-26000.142665* and *Cluster-26000.124336* (SA-related) were significantly up-regulated at PI stage, inferring they might play key roles during female organ development in *A. amurensis*.

**Fig. 7:**
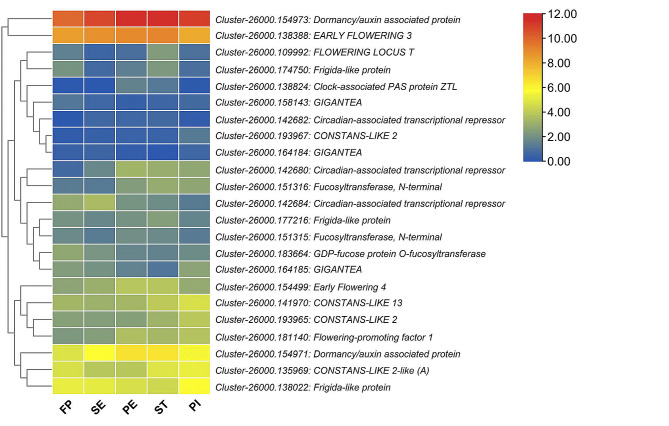
Heatmap of log_2_ FC values of the DEGs related to circadian, photoperiod, and flowering

To better understand flowering mechanisms in *A. amurensis*, we screened out differentially expressed circadian and flowering-related genes. In total, 23 genes were identified, and their expression patterns are shown in Fig. 7. They included one *FLOWERING LOCUS T* (*Cluster-26000.109992*), four *CONSTANS*-like genes (*Cluster-26000.193967, Cluster-26000.141970, Cluster-26000.193965*, and *Cluster-26000.195969*), three *GIGANTEA* genes (*Cluster-26000.158143, Cluster-26000.164184*, and *Cluster-26000.164184*), three Frigida-like genes (*Cluster-26000.174750, Cluster-26000.177216*, and *Cluster-26000.138022*), three circadian-associated transcriptional repressors (*Cluster-26000.142682, Cluster-26000.142680*, and *Cluster-26000.142684*), one *EARLY FLOWERING 3* (*Cluster-26000.138388*), two dormancy-related genes (*Cluster-26000.154973* and *Cluster-26000.154971*), etc. Compared to other genes, *Cluster-26000.138388* (*ELF3*) and *Cluster-26000.154973* (dormancy-related) were highly induced along with the flower organogenesis (Fig. 7).

Three CONSTANS-like genes (*Cluster-26000.193967, Cluster-26000.141970*, and *Cluster-26000.193965*), were specifically up-regulated (|log2Fold Change| > 1.3) at PI stage. Among the circadian-associated transcriptional repressors, *Cluster-26000.142680* was up-regulated from the PE stage, while *Cluster-26000.142684* was down-regulated at the ST stage. *Cluster-26000.142682* was specifically up-regulated at the ST stage. *Cluster-26000.138824*, encoding a Clock-associated PAS protein ZTL, was specifically up-regulated (|log_2_ Fold Change| > 4.2) at PE and ST stages. The genes *FT* (*Cluster-26000.109992*) and one *Frigida*-like (*Cluster-26000.174750*) were down-regulated except at FP and ST stages. The gene *Cluster-26000.181140*, which encodes a Flowering-promoting factor 1 (*FPF1*), was up-regulated, while the *GIGANTEA* genes were down-regulated along with the flower differentiation.

Besides, two DEGs, *Cluster-26000.122897* and *Cluster-26000.47006*, identified in the comparison between FP_vs_SE and SE_vs_PE, respectively, were enriched in vernalization response, suggesting they might play important roles in the regulation of *A. amurensis* flowering. *Cluster-26000.122897* encodes a *VERNALIZATION INDEPENDENCE 4*, while *Cluster-26000.47006* was not annotated. The two genes showed contradictory expression patterns along with flower differentiation. *Cluster-26000.122897* was down-regulated, while *Cluster-26000.47006* was up-regulated, suggesting *Cluster-26000.47006* might regulate the expression of *Cluster-26000.122897* during the underground flower differentiation process in *A. amurensis*.

### Candidate genes and qRT-PCR validation

The above results indicated that TFs, phytohormones, and some flowering-related genes play critical roles during flower differentiation in *A. amurensis*. Therefore, based on the gene expression fold changes, we selected 186 genes as candidate genes for future functional studies to decipher the regulatory network of flower development in *A. amurensis* (Table S8).

**Fig. 8:**
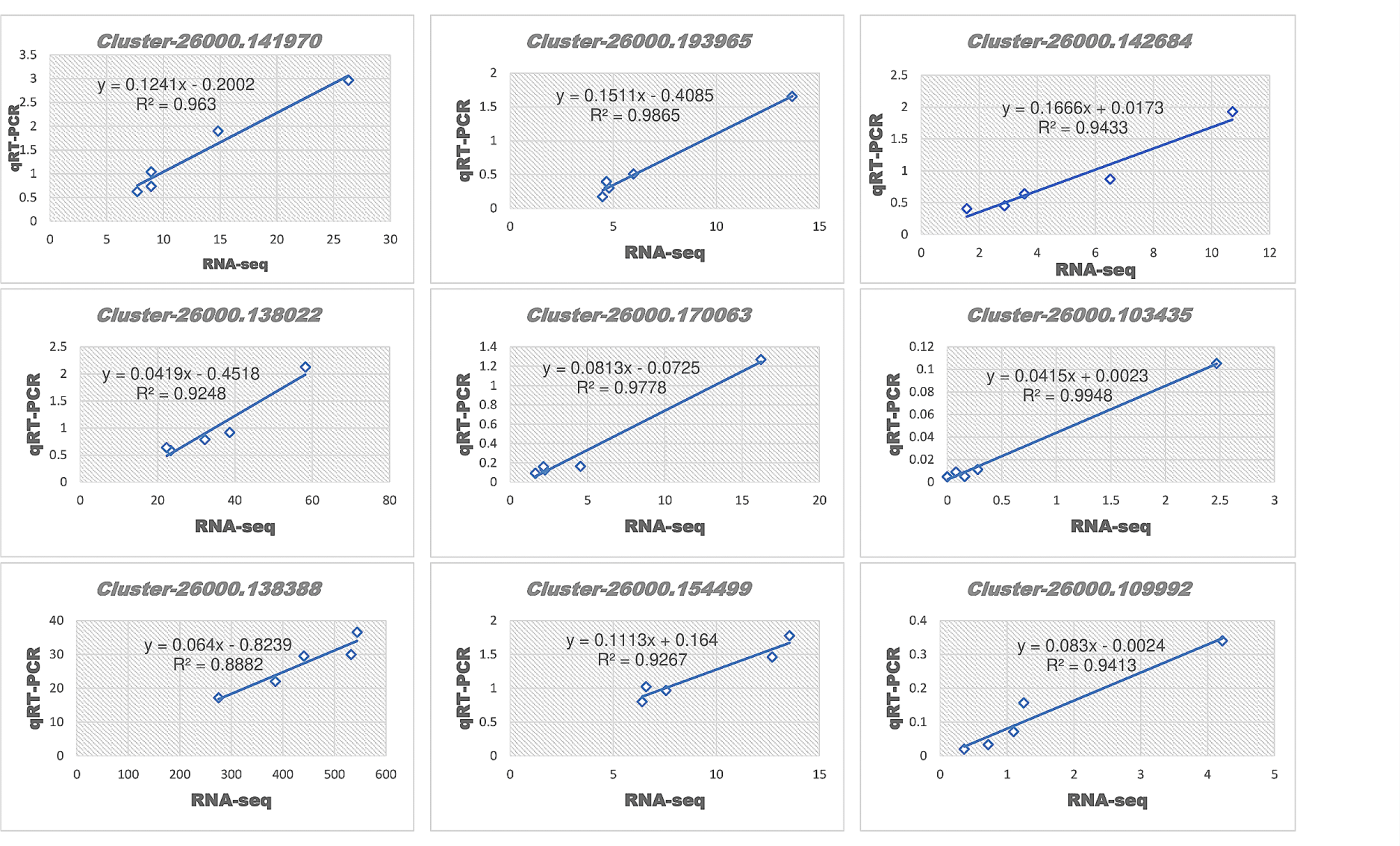
Validation of the expression of nine randomly selected DEGs via qRT-PCR.

To validate our results, we randomly selected nine DEGs for qRT-PCR analysis. There is no defined rule on choosing genes for qRT-PCR. Different research groups have selected different number of genes [31–34]. The expressions of the genes via qRT-PCR were consistent with that of the RNA-seq data, with a correlation coefficient of *r* > 0.88 (Fig. 8). These results confirm our findings are reliable.

## Discussion

Knowledge of molecular mechanisms governing reproductive growth in understory species that complete their lifecycle in particular environments is of great interest to improve the general understanding of the complex genetic regulatory network of the flowering process in Angiosperm plants. This study characterized flower organogenesis and unveiled key regulatory pathways and genes in *A. amurensis*, a potential ornamental ephemeral spring plant, through microscopic observations and comparative transcriptomics analysis of meristems at different developmental stages. Transcriptome sequencing and analysis have been widely applied to insight into the regulation of flower development in diverse species, such as *Erythronium japonicum* Decne [17], *Arundina graminifolia* [35, 36], *Populus tomentosa* [13], blueberry [37], and *Vicia sativa* [38].

The switch from vegetative growth to the reproductive growth of Angiosperm plants is generally affected by environmental factors, mainly ambient temperature and photoperiod [5, 12, 39]. However, in *A. amurensis*, the whole floral differentiation process occurs underground without photoperiod and vernalization, implying that these pathways might not regulate flower organogenesis. Supportively, our analyses revealed that the major photoperiod and vernalization pathway genes, including *CO-likes*, *GI*, and *VIN4*, were mainly down-regulated during the floral differentiation. Moreover, *ELF3*, a *GI* repressor, and some genes encoding circadian-associated transcriptional repressor were significantly up-regulated. *GI* promotes the expression of *CO* and *FT*, while *ELF3* induces *GI* degradation [5, 12, 40, 41]. *VIP4* confers loss of *FLC* expression and early flowering in the absence of cold [42]. Therefore, *VIP4* down-regulation during floral differentiation is in accordance with *A. amurensis* plant phenology. The gene *FPF1* was up-regulated along with the flower organogenesis. *FPF1* is a critical gene in the gibberellin pathway that modulates the acquisition to flower in apical meristems [43, 44]. Together, these results indicate that the gibberellin pathway might be the major driven pathway of flower organogenesis in *A. amurensis*.

DEGs analysis revealed that the floral differentiation in *A. amurensis* is stage-specific regulated, and the key regulators are phytohormones and TFs. These findings are consistent with reports in *E. japonicum*, which has the same reproductive phenology [17]. *FT* (*Cluster-26000.109992*) and one *FRI*-like (*Cluster-26000.174750*) were down-regulated except at FP and ST stages, indicating they might play important roles during the floral differentiation. *FT* might modulate the flower organogenesis process under the control of phytohormones and TFs [12]. Three *CO*-like genes (*Cluster-26000.193967*, *Cluster-26000.141970*, and *Cluster-26000.193965*) were specifically up-regulated at the PI stage. These *CO*-like and *FRI-*like genes might cooperate to repress flowering to occur before early spring. *FRI* genes are known to promote *FLC* expression, the master repressor of flowering [12, 45]. In rice, *OsCOL15* (*CONSTANS-LIKE 15*) suppresses flowering by activating *Ghd7* and *RID1* [46]. The genes *Cluster-26000.154973 and Cluster-26000.154971* that encode dormancy/auxin-associated protein were induced along with the flower differentiation, suggesting they might be the master regulators of underground dormancy. Moreover, these genes may also be involved in flowering repression. We identified several auxins, brassinosteroid, salicylic acid, ABA, gibberellin, jasmonic acid, and cytokinin-related DEGs that might play essential regulatory functions during flower ‘organs’ development in *A. amurensis*. The functions of phytohormones in regulating reproductive processes have been widely studied and discussed [1, 11, 47, 48].

Previous studies have demonstrated that many TF family genes play critical roles in regulating the flowering process in *A. amurensis* [18–20]. However, their involvement in underground flower organogenesis was not analyzed. In this study, we found that diverse TFs, mostly MADS, FAR1, MYBs, AP2/ERF, B3, C2H2, LOB, and GRAS (DELLA) family genes were significantly up- or down-regulated depending on the floral differentiation stage, indicating there are main components of the regulatory network of the underground flower organogenesis in *A. amurensis*. Similar results were reported in *E. japonicum* [17]. Furthermore, the roles of TFs in flower organogenesis have been analyzed in many species [12, 17, 49, 50]. Among these TFs, DELLA proteins might play central roles through their multilevel inter-regulation patterns with gibberellin [51, 52]. We have identified 186 candidate genes, including TFs, phytohormones, and circadian/flowering-related genes. Functional characterization of these genes is needed to understand the complex regulatory network of the underground flower organogenesis in *A. amurensis*. This will help identify key gene markers for optimizing the ‘plant’s production and enhancing its ornamental value in the current global climate change situation [53, 54].

In our comprehensive analysis of the *A. amurensis* flower during development, we observed distinct gene expression patterns associated with consecutive developmental stages. Our data reveal a complex network of DEGs predominantly engaged in pathways critical for flower development and maturation. Notably, these DEGs were significantly enriched in the biosynthesis of secondary metabolites, protein processing in the endoplasmic reticulum, and plant hormone signal transduction, aligning with previous reports that underscore the pivotal role of these pathways in flower organogenesis and function [55, 56]. Additionally, our results highlight the involvement of starch and sucrose metabolism, ABA transporters, and the MAPK signaling pathway in floral development, corroborating Wu et al.‘s findings, where they treated the plants with cold stress and performed the transcriptome analysis [57]. Similar results were obtained by another research group who treated the *Argyranthemum frutescens* with cold stress [58]. Our targeted analysis of flowering-related gene ontology (GO) terms further facilitated a granular view of the regulatory landscape governing floral organ development. Our data shows that the transition from vegetative to reproductive phase (ST to PI) marks a critical window characterized by heightened transcriptional activity, echoing the conclusions of other researchers who studied stage-specific gene expression during flower morphogenesis [59]. In stark contrast, the PE to ST transition appears transcriptionally subdued, suggesting a potential preparatory phase where transcriptional changes are minimal. Among the flowering-related GO terms, our study highlighted several key processes overwhelmingly represented during these transitions. These include the vegetative to reproductive phase transition of meristem, flower whorl development, flower organ development, embryo sac central cell differentiation, pollen tube growth and development, and regulation of flower development. These findings are in harmony with the work of others, who also reported these processes as central to flower development and maturation [60].

## Conclusions

This study explored the molecular changes that occur during the underground floral differentiation in *A. amurensis* through transcriptomics analysis of meristems at five different developmental stages. Through DEGs analysis and functional annotation, we found that TF family genes and phytohormones may co-regulate different pathways involved in flower organogenesis in *A. amurensis*. We identified 186 potential candidate genes, including hormone-related, flowering/circadian-related, and TF family genes that should be functionally characterized in future studies to decipher the complex genetic regulatory network underlying the underground floral differentiation process in *A. amurensis* and other spring ephemeral species.

## Potential directions for future studies

In our study, we identified candidate genes, which also play a role as TFs, including hormone-related, flowering/circadian-related, and TF family genes during the underground floral differentiation process in Adonis amurensis. These findings provide a valuable resource for future functional characterization to understand this species’ complex genetic regulatory network and other spring ephemerals. In conclusion, our study provides a foundation for a wide range of future research endeavors that could significantly advance our understanding of plant biology, particularly in relation to flowering processes in unique ecological contexts.

### Electronic supplementary material

Below is the link to the electronic supplementary material.

Supplementary Material 1

Supplementary Material 2

## Data Availability

The raw data of the RNA-seq is available at NCBI SRA under the project number: PRJNA730644.
