# Supplementary material for: Dynamic transcriptome analysis provides molecular insights into underground floral differentiation in Adonis Amurensis Regel & Radde
Source: BMC Genom Data. 2024 Mar 21;25:33. doi: 10.1186/s12863-024-01220-2 (PMC10956236; doi:10.1186/s12863-024-01220-2)
Supplement: Supplementary file 2 — Supplementary Material 2 [file 12863_2024_1220_MOESM2_ESM.pdf]

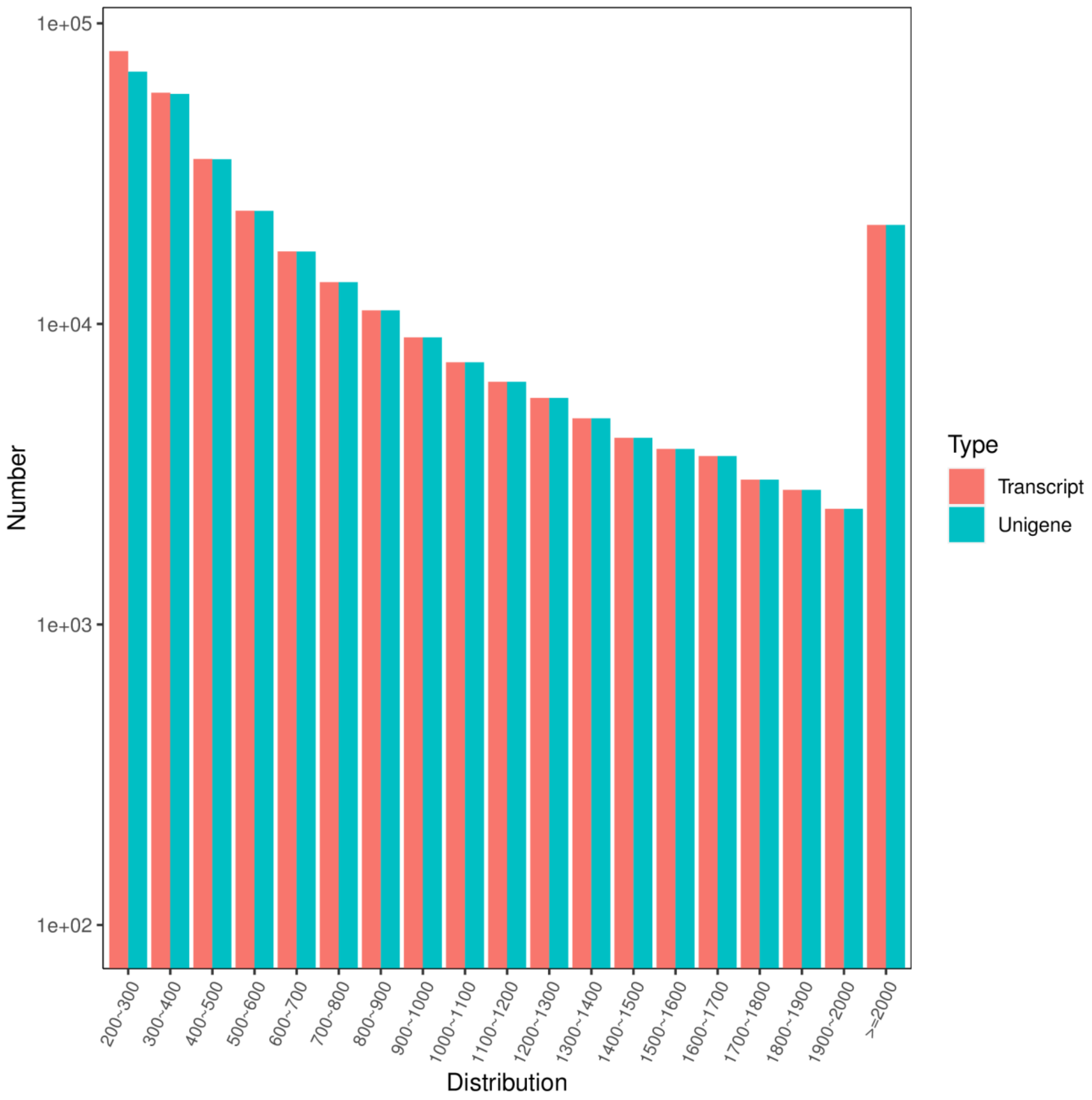

Figure S1. Distribution of the Length of transcripts and unigenes.

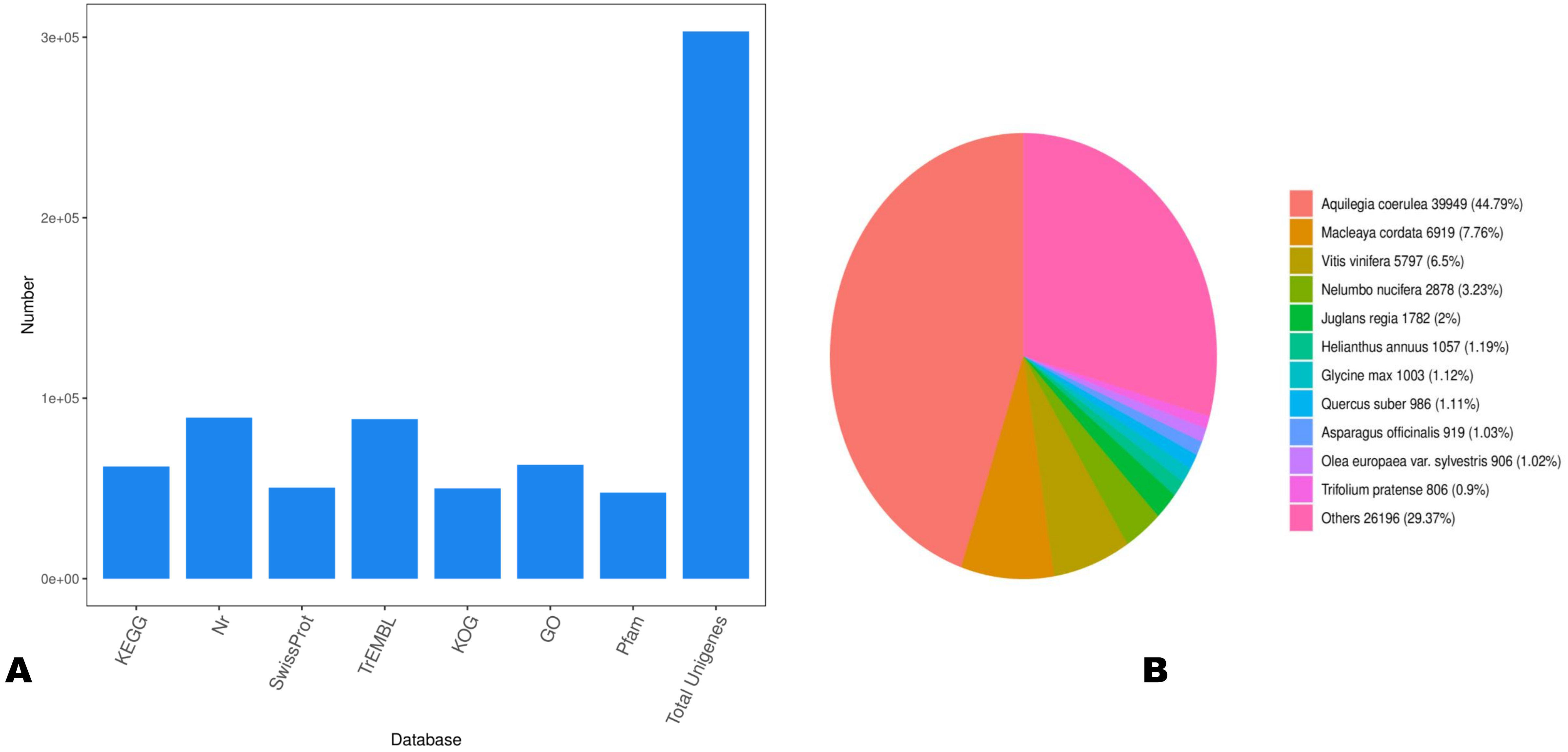

Figure S2. (A) Mapping rates of unigenes against different databases. (B) Similarity result of unigenes with sequences in other species.

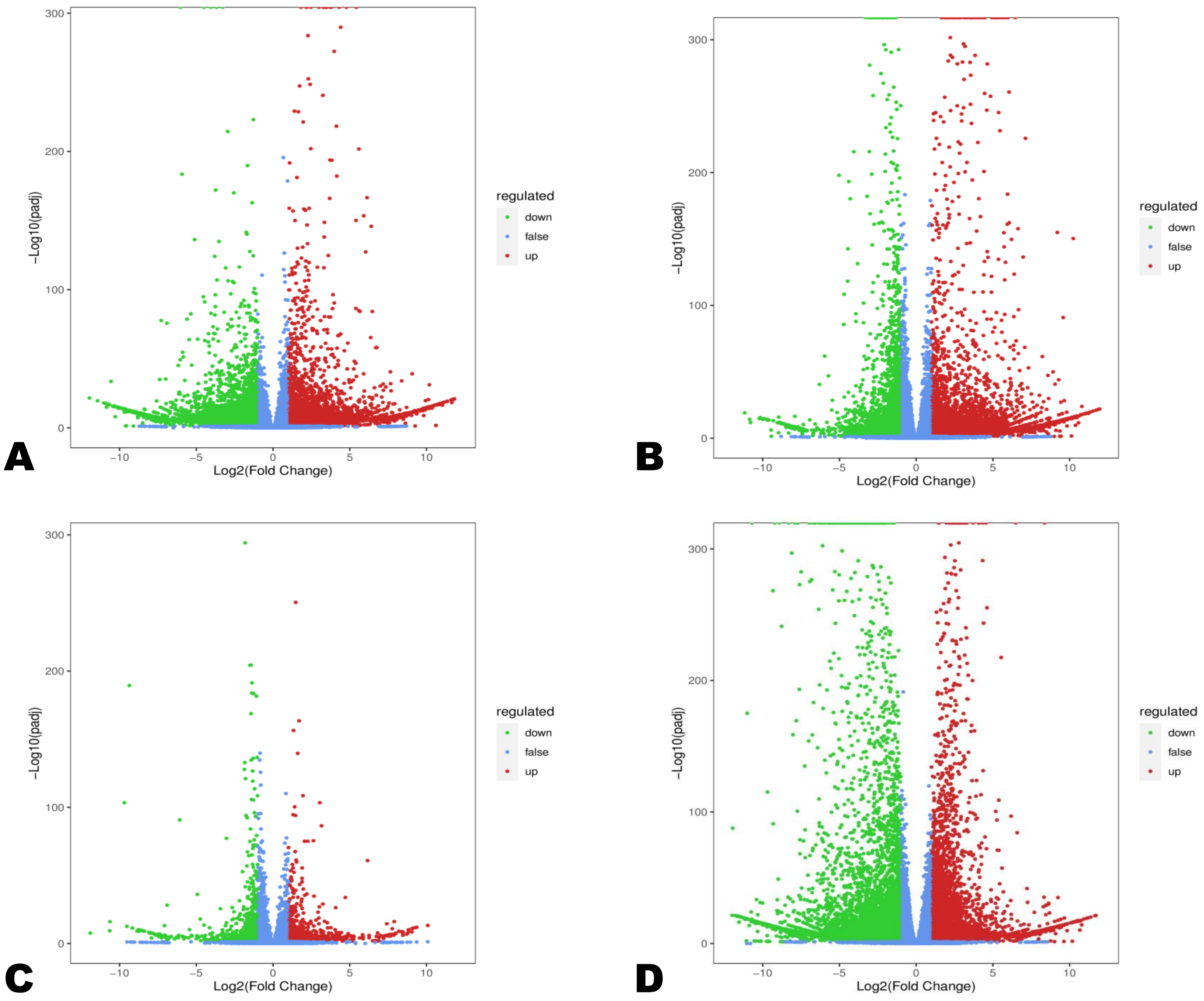

Figure S3. Volcano plots of DEGs in the pairwise comparison between: (A) FP and SE; (B) SE and PE; (C) PE and ST; (D) ST and PI.

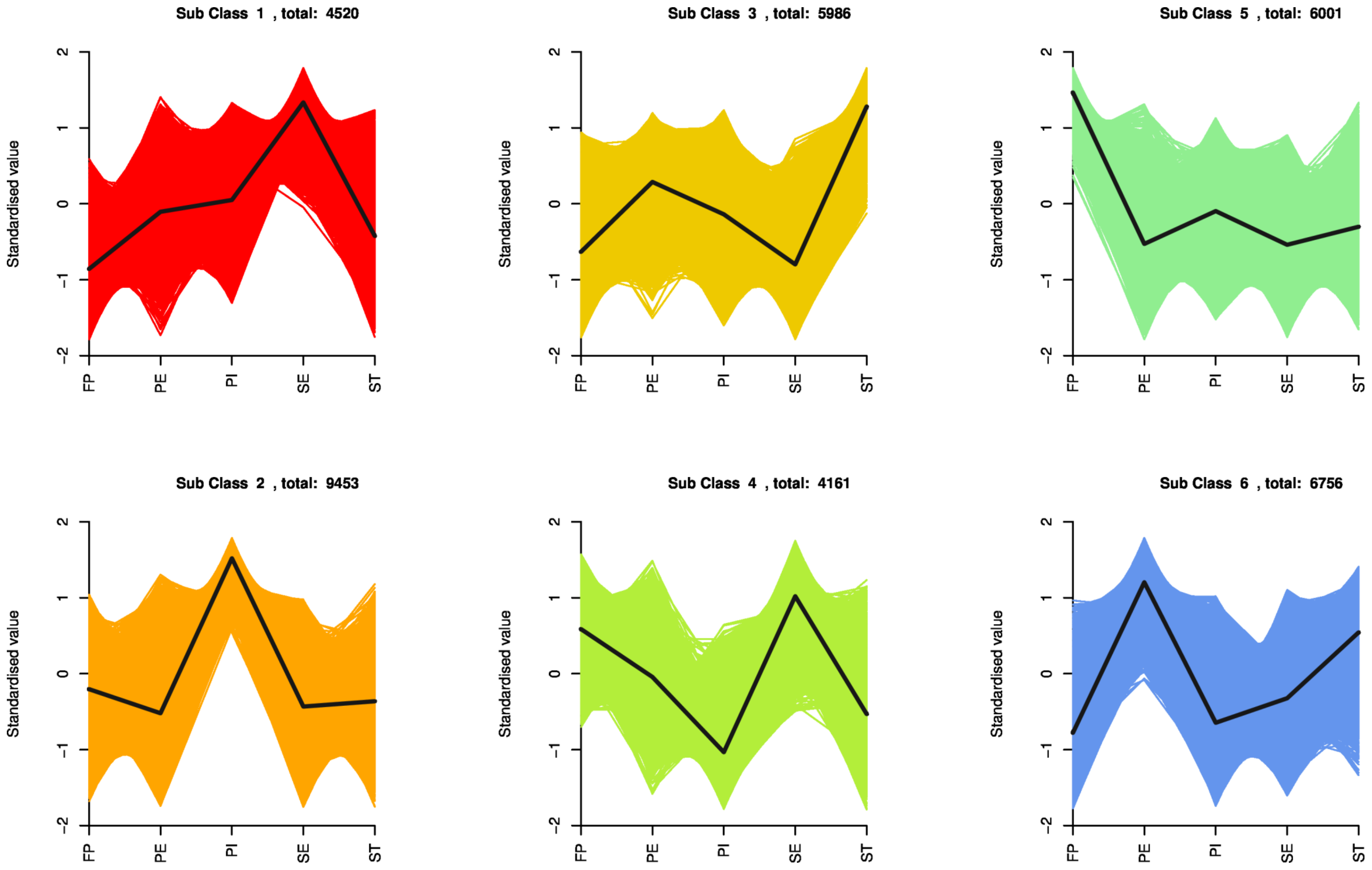

Figure S4. Kmeans analysis result of DEGs.

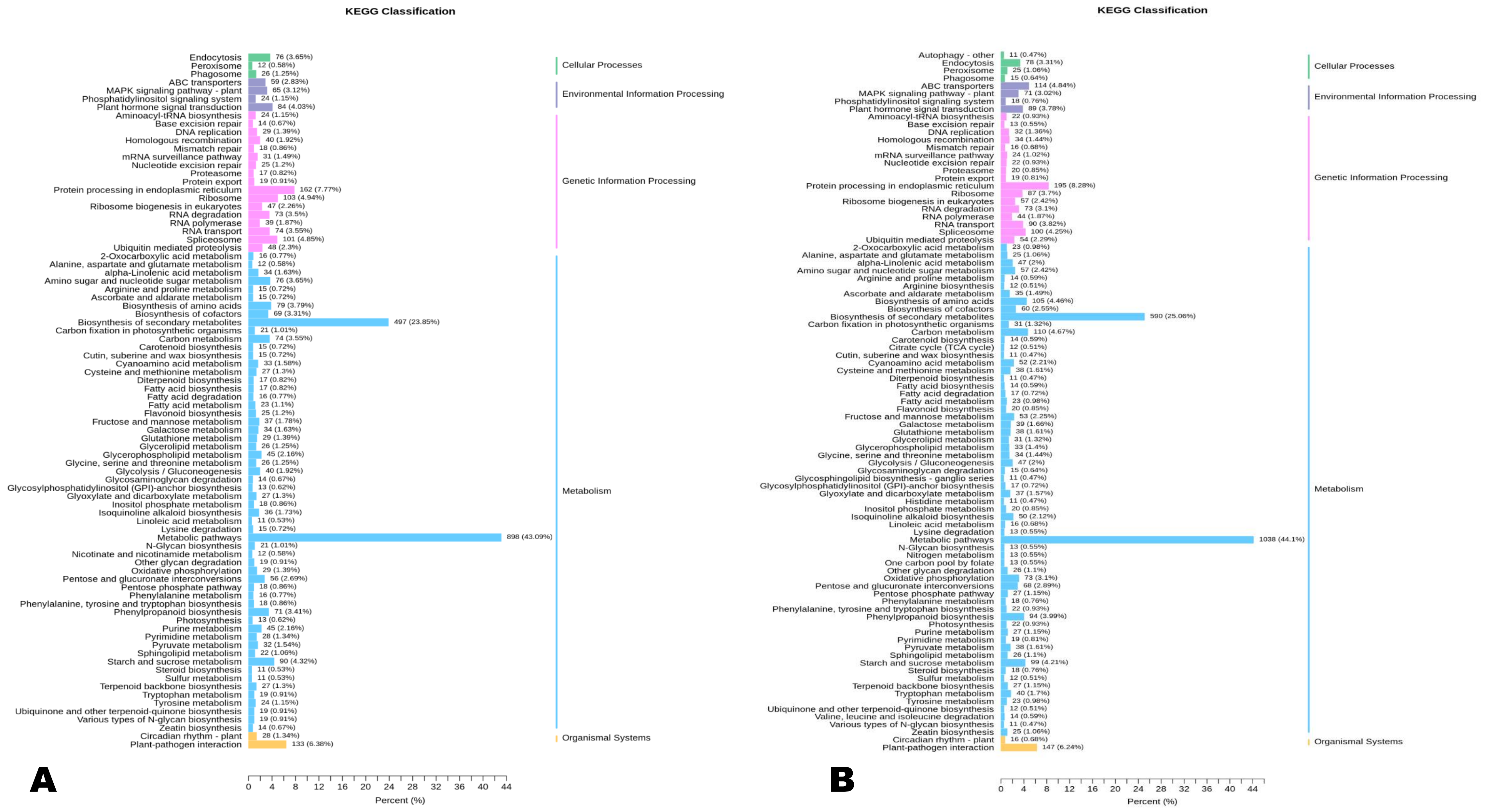

Figure S5. KEGG analysis results of DEGs in the pairwise comparison between: (A) FP and SE; (B) SE and PE.

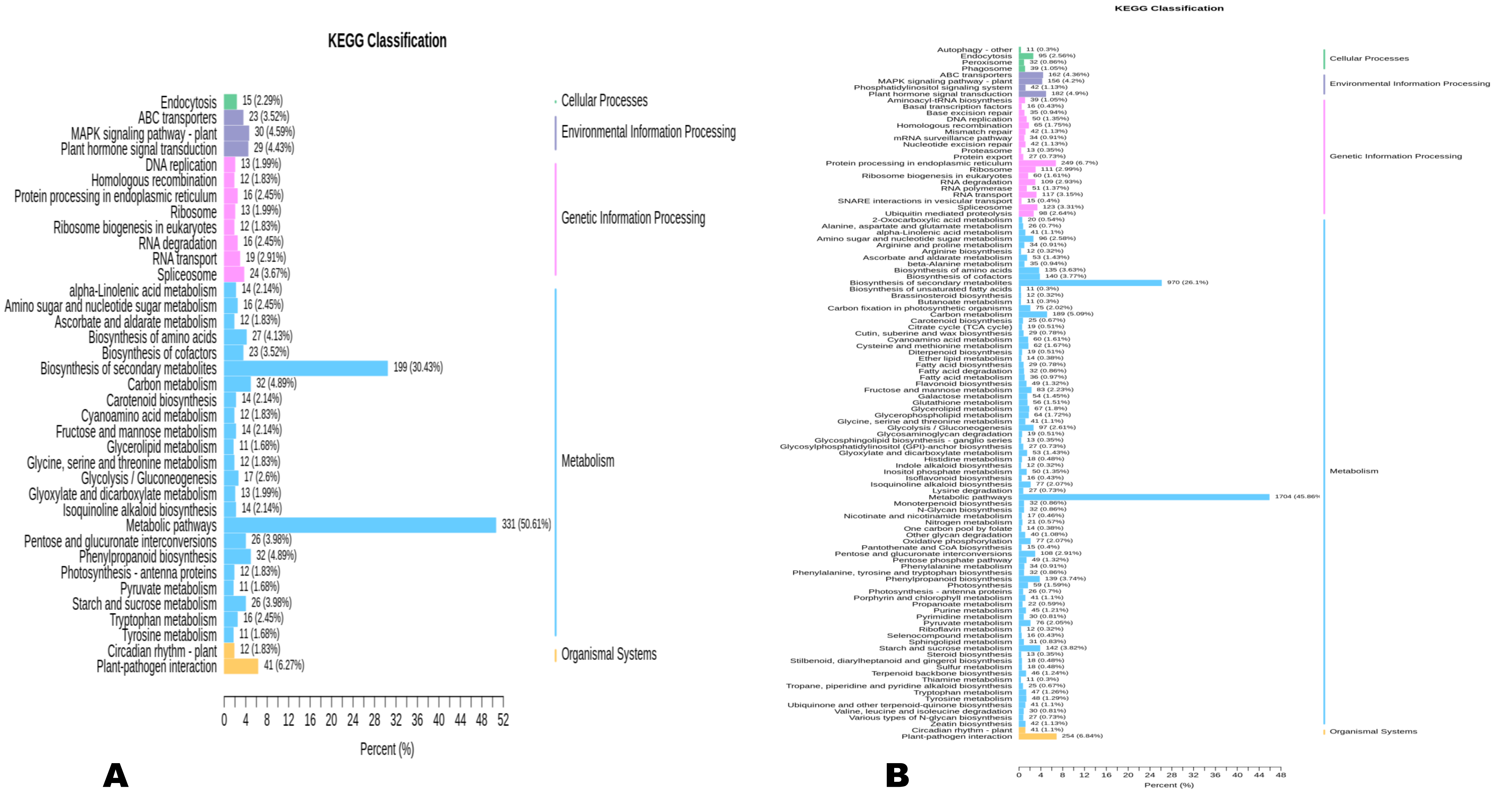

Figure S6. KEGG analysis results of DEGs in the pairwise comparison between: (A) PE and ST; (B) ST and PI.

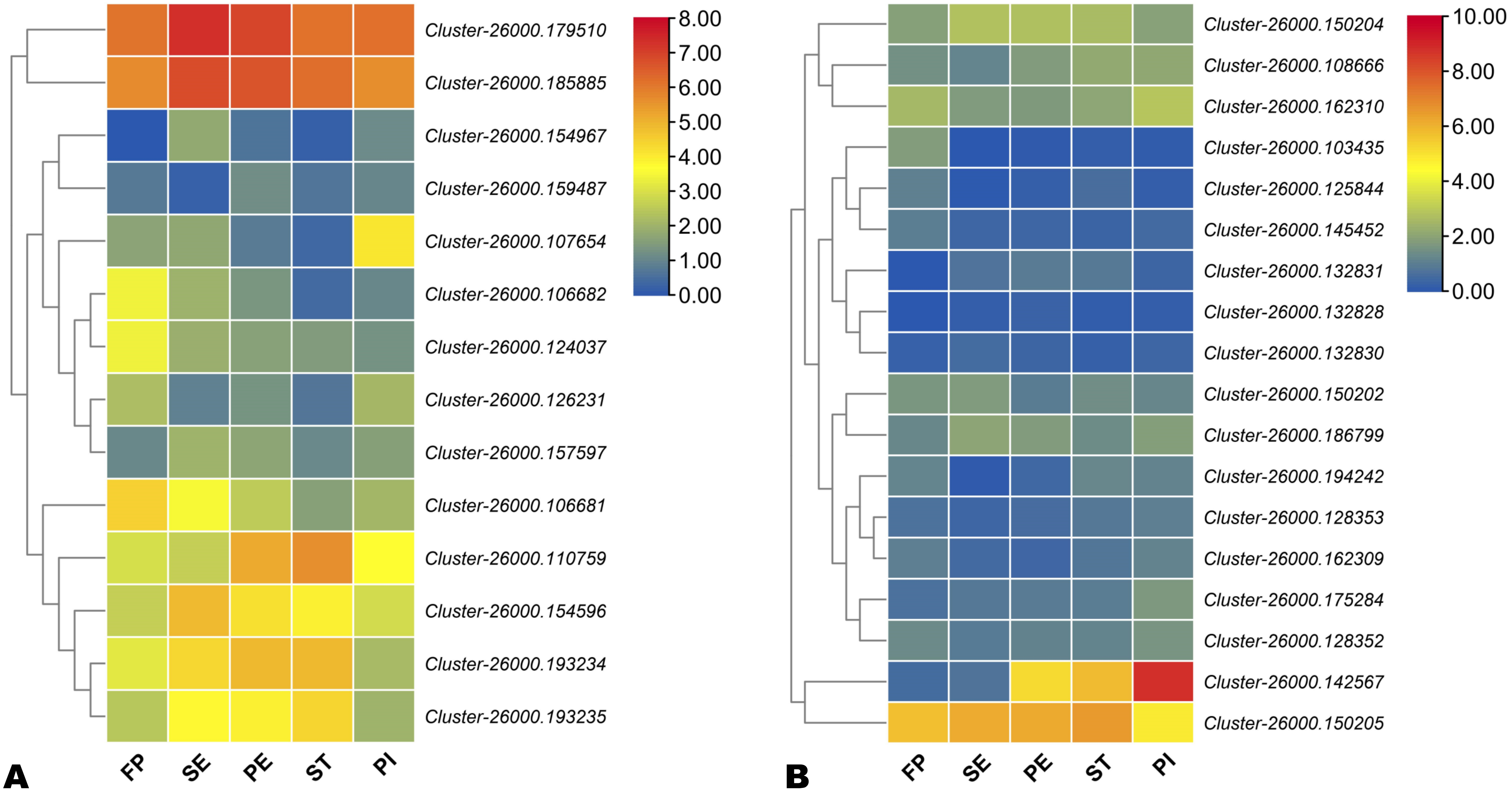

Figure S7. Heatmap of log2FC values of the DEGs belonging to EP2/ERF (A) and LOB (B) families.

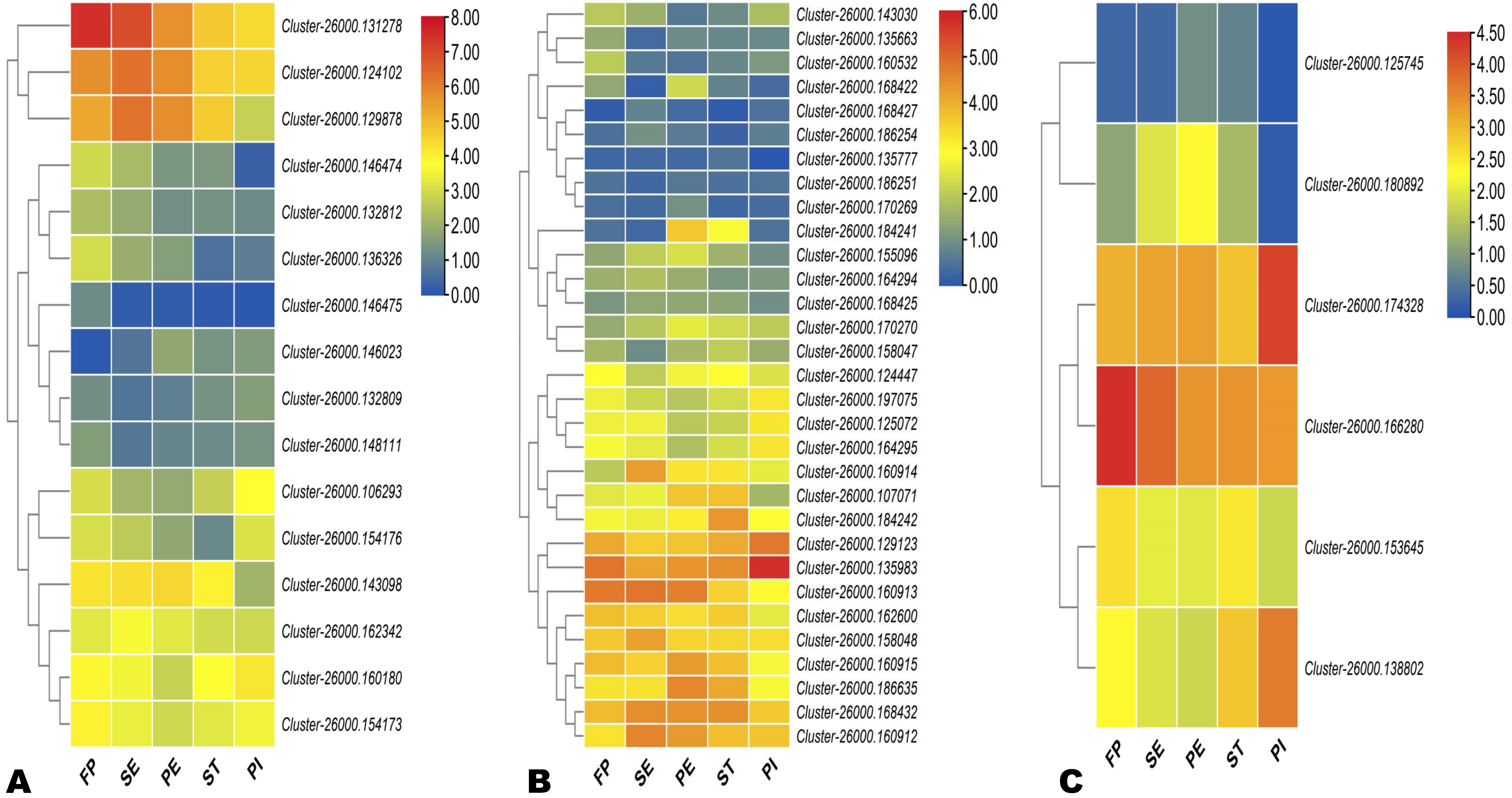

Figure S8. Heatmap of log2FC values of phytohormone-related DEGs. (A) ABA. (B) Brassinosteroids. (C) Cytokinin.
